# Nitrogen use efficiency is regulated by interacting proteins relevant to development in wheat

**DOI:** 10.1111/pbi.12864

**Published:** 2018-01-15

**Authors:** Lei Lei, Genqiao Li, Hailin Zhang, Carol Powers, Tilin Fang, Yihua Chen, Shuwen Wang, Xinkai Zhu, Brett F. Carver, Liuling Yan

**Affiliations:** ^1^ Department of Plant and Soil Sciences Oklahoma State University Stillwater OK USA; ^2^ Present address: Wheat, Peanut and Other Field Crops Research Unit USDA‐ARS Stillwater OK USA; ^3^ Present address: The Land Institute Salina KS USA; ^4^ Present address: Key Laboratory of Crop Genetics and Physiology of Jiangsu Province Yangzhou University Yangzhou Jiangsu China

**Keywords:** nitrogen use efficiency (NUE), *Ta* VRN‐A1, *Ta* ANR1, *Ta* HOX1, flowering time, wheat

## Abstract

Wheat (*Triticum aestivum*) has low nitrogen use efficiency (NUE). The genetic mechanisms controlling NUE are unknown. Positional cloning of a major quantitative trait locus for N‐related agronomic traits showed that the vernalization gene *TaVRN‐A1* was tightly linked with *TaNUE1*, the gene shown to influence NUE in wheat. Because of an Ala^180^/Val^180^ substitution, *Ta* VRN‐A1a and *Ta* VRN‐A1b proteins interact differentially with *Ta* ANR1, a protein encoded by a wheat orthologue of *Arabidopsis nitrate regulated 1* (*ANR1*). The transcripts of both *TaVRN*‐*A1* and *TaANR1* were down‐regulated by nitrogen. *TaANR1* was functionally characterized in *TaANR1::RNAi* transgenic wheat, and in a natural mutant with a 23‐bp deletion including 10‐bp at the 5′ end of intron 5 and 13‐bp of exon 6 in gDNA sequence in its gDNA sequence, which produced transcript that lacked the full 84‐bp exon 6. Both *Ta* ANR1 and *Ta* HOX1 bound to the Ala^180^/Val^180^ position of *Ta* VRN‐A1. Genetically incorporating favourable alleles from *TaVRN‐A1*,* TaANR1* and *TaHOX1* increased grain yield from 9.84% to 11.58% in the field. Molecular markers for allelic variation of the genes that regulate nitrogen can be used in breeding programmes aimed at improving NUE and yield in novel wheat cultivars.

## Introduction

Nitrogen (N) is the most important nutrient for plant development and growth, and soil is often supplemented with N fertilizer to ensure successful seed production and high grain yield for non‐N‐fixing food crops such as wheat (*Triticum aestivum* L.), rice (*Oryza sativa* L.) and maize (*Zea mays* L.) (Santi *et al*., 2013). A sevenfold increase in the use of N fertilizer was found to be associated with a twofold increase in food production over the past four decades (Hirel *et al*., 2007; Shrawat *et al*., 2008). Given the projected increase in the world's human population to over 9 billion by 2050, a further threefold increase in N input is expected to be needed to meet the world's demand for major crop products (Cormier *et al*., 2013; Schroeder *et al*., 2013).

Although N fertilizer has the most direct and efficient approach for increasing crop production, the synthetic N fertilizers supplied to soils have immediate and adverse effects on the environment and climate. Only 30%–35% of added N fertilizers are taken up and used by wheat plants in the year of application, and the remaining 65%–70% (assuming fertilizer–soil equilibrium) is lost, predominantly as nitrous oxide, through gaseous plant emission, soil denitrification, surface run‐off, volatilization and leaching, which contributes to atmospheric greenhouse gases and environmental pollution (Gaju *et al*., 2011; Raun and Johnson, 1999). Developing varieties of wheat that require less N input yet maintain the same or higher grain yields is an economically and environmentally sustainable goal in international agriculture.

The response of plants to added N involves genes in several pathways, including uptake, assimilation and translocation, as well as recycling and remobilization of N within the plants, and these responses differ according to genotype, environment, N level and plant age (Krapp, 2015). Wheat is traditionally divided into two types: winter wheat that requires the plant to be exposed to low temperatures during a winter season to accelerate its transition from vegetative to reproductive development (vernalization), and spring wheat, which has no requirement for vernalization (Pugsley, 1971). Compared to spring wheat, winter wheat requires significantly more N to achieve maximum grain yield, because it has a longer growing season with greater potential for leaching, volatilization and run‐off losses (Goos and Johnston, 1999). Dual‐purpose winter wheat cultivars planted in the Southern Great Plains (USA) require more N, because N is removed in grazed forage (MacKown and Carver, 2007). Thus, it is of interest to determine which genes regulate nitrogen use efficiency (NUE) when limited N fertilizers are supplied to winter wheat. Multiple genes involved in the response to N that influence root/shoot growth, N metabolism and N content have been identified in *Arabidopsis thaliana*. The N‐related genes are divided into two classes: those involved in growth, such as *Arabidopsis nitrate regulated 1* (*ANR1*) (Zhang and Forde, 1998) and *superroot* (Boerjan *et al*., 1995), and those involved in nitrogen metabolism pathways, such as the *SAT1* transporter for initial ammonia uptake (Kaiser *et al*., 1998), *CHL2* for nitrate reductase (LaBrie *et al*., 1992) and genes encoding three enzymes involved in ammonium assimilation ((i.e. glutamine synthetase (GS) (Peterman and Goodman, 1991), glutamate synthase (*GLU1*) and glutamate dehydrogenase (*GDH1*) (Lam *et al*., 1995)). Other genes involved in the reduction of nitrate to nitrite, and the subsequent reduction of nitrite to ammonium, include nitrate transporter (*NRT*) (Tsay *et al*., 1993) and nitrite reductase (*NiR*) (Leydecker *et al*., 2000). In rice, heterotrimeric G proteins were found to regulate NUE (Sun *et al*., 2014). In wheat, *TaNAC2‐5A*, a transcription factor encoding a NAC (NAM, ATAF and CUC), was found to bind to the promoter region of the genes encoding nitrate transporter and glutamine synthetase, resulting in an enhanced ability of roots to acquire nitrogen and increased grain yield (He *et al*., 2015). *TaNFYA‐B1*, a subunit of nuclear factor Y, stimulated root development and up‐regulated the expression of the genes encoding nitrate transporters in roots, resulting in a significant increase in nitrogen uptake and grain yield (Qu *et al*., 2015).

In Arabidopsis, quantitative trait loci (QTLs) mapping is an efficient method to determine which genomic regions contribute to NUE in different N environments (Loudet *et al*., 2003; Rauh *et al*., 2002). Hundreds of wheat QTLs or genomic regions are associated with N‐related agronomic traits, but each accounts for only a small part (<25%) of the total phenotypic variation (An *et al*., 2006; Cormier *et al*., 2014; Habash *et al*., 2007; Mickelbart *et al*., 2015; Quarrie *et al*., 2005; Xu *et al*., 2014), which has limited molecular cloning of the mapped QTLs. A glutamate synthase (GoGAT) gene on wheat chromosome 3B contributed to NUE in wheat based on physical mapping, sequence analysis and functional validation of an NUE MetaQTL (Quraishi *et al*., 2011). In the previous study, the *TaVRN‐A1* locus on chromosome 5A was mapped to colocate with a QTL for NUE in wheat, but it was unknown how this locus contributed to NUE in wheat. In this study, we evaluated a population of recombinant inbred lines (RILs) of winter wheat under contrasting N fertilization regimes and identified a major QTL for N‐related agronomic traits. We cloned the gene associated with this QTL and further identified interactors of the protein encoded by the cloned gene.

## Results

### Genetic identification of a major QTL for NUE

Two populations from the same 96 RILs and the parental lines ‘Jagger’ and ‘2174’ were initially grown in a greenhouse on Kirkland silt loam soil in which N was severely deficient but other macronutrients were sufficient (Table S1), under long‐day conditions and constant temperature, but without vernalization (Figure S1a). Under such conditions, the winter wheat plants remained in the vegetative stage and showed similar phenotypes until 11 weeks after planting (Figure S1b). When plants were starved of N, two different levels of N, 14 mg N kg^−1^ soil (25 N, equivalent to 25 kg N ha^−1^) and 56 mg N kg^−1^ soil (100 N, equivalent to 100 kg N ha^−1^), were used to fertilize the plants. Parental lines started to show differences in their morphological traits 3 weeks after N fertilization (Figure S1c).

**Figure 1:**
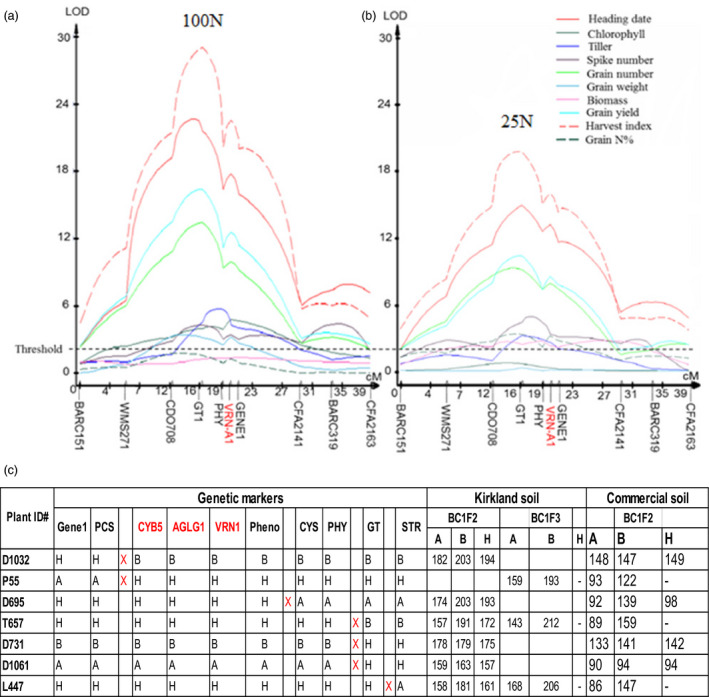
Genetic mapping and phenotypic effects of *QNue.osu‐5A*. Two sets of the Jagger × 2174 recombinant inbred line (RIL) population were evaluated in a temperature‐ and photoperiod‐controlled greenhouse, and in Kirkland soil that was N‐deficient but adequate in other essential nutrients for 11 weeks. Then, two different levels of N fertilizer (25N and 100N) were supplied to the plants. (a) QTLs for N‐related traits under the 100N condition. The *TaVRN‐A1* gene on chromosome 5AL is highlighted in red. (b) QTLs for N‐related traits under the 25N condition. The horizontal dashed line represents a threshold value of 2.5 log of the odds (LOD) for N‐related traits. (c) Phenotypes (heading dates (days from planting)) and genotypes of seven critical recombinant lines with crossovers within the *QNue.osu‐5A* locus. ‘A’, the Jagger allele; ‘B’, the 2174 allele; and ‘H’, the heterozygous state. Pheno represents phenotypes. Three candidate genes are highlighted in red. ‘X’ indicates a crossover between two neighbouring markers.

As the N‐fertilized populations were grown under the same, continuous greenhouse conditions until they reached maturity, 10 N‐related traits were phenotyped. A major QTL on the long arm of chromosome 5A was mapped associated with the N‐related traits, but the colocalized QTLs largely differed in the log of the odds (LOD) for magnitudes of the total variation between the two contrasting N regimes (Figure 1a, b; Table S2). Furthermore, statistical analyses showed that interactions of the QTLs with N rate were significant or highly significant for five traits including heading date, leaf chlorophyll content, grains per spike, grain yield per plant and harvest index (Table S3). Therefore, these five traits should be regulated by the gene at the QTL. In the greenhouse study, winter wheat plants were not vernalized, to avoid the effect of vernalization; hence, these plants showed large variations in heading date. Those lines carrying the Jagger allele had an earlier average heading date and produced more grains, but lines carrying the 2174 allele showed delayed heading and produced fewer grains. The average grain yield of those lines carrying the Jagger allele was 0.84 g per plant, an increase of 140% compared with 0.35 g per plant for lines carrying the 2174 allele, when plants were grown in the same 100 N soil (Table S2). These results indicated that the N‐regulated gene at the QTL affected development and thus grain yield. The QTL on wheat chromosome 5AL for the multiple N‐related traits was temporarily designated *QNue.osu‐5A*, and the gene causing the QTL was designated *TaNUE1*, the gene shown to have pleiotropic effects in hexaploid wheat.

To determine which gene is *TaNUE1*, fifteen recombinant events that occurred at *QNue.osu‐5A* were used to fine map the QTL in the Kirkland soil and in the same greenhouse with long days and constant temperatures as used to find *QNue.osu‐5A* (Table S4). These recombinant lines were generated from 6410 BC_1_F_3_ plants that were used to clone the gene responsible for vernalization requirement duration in winter wheat in our previous study (Li *et al*., 2013). PCR markers for the allelic variation of each gene were developed and mapped in the RILs (Figure S2). Seven of these recombinant lines were selected for testing in the commercial soil. Based on the segregation of heading date in these recombinant lines (Figure 1c; Table S4), the location of *TaNUE1* was narrowed down to a region containing three candidate genes, *TaCYB5*,* TaAGLG1*, and *TaVRN‐A1* (Figure 1c).

**Figure 2:**
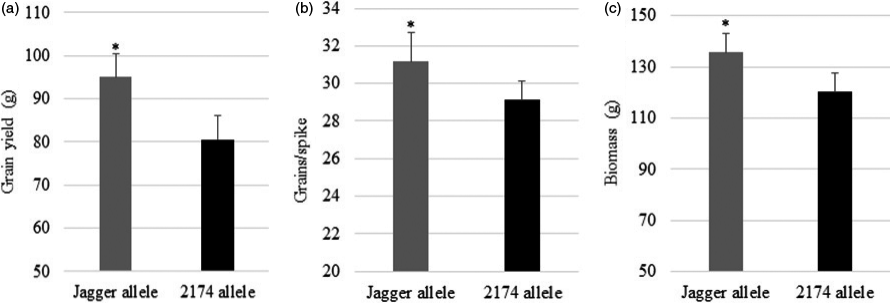
Phenotypic effects of *TaNUE1* in the field. Several critical recombinant lines were tested in a field at Oklahoma State University Cimarron Valley Research Station in the 2011–2012 growing season. (a) Grain yield. (b) Grains per spike. (c) Biomass. The average values of each genotype in each critical recombinant line were compared, and the bars indicate standard errors. n = 6 for the Jagger allele, and n = 8 for the 2174 allele. Asterisk indicates that the difference was significant between the two alleles (*Pr* < 0.05).

The seven recombinant lines were also tested in a field with Teller loam soil with 7.5 ppm NO_3_^−^ N, representing an N‐stress condition (Table S1). As shown in Figure 2, plants carrying the Jagger *TaNUE1* allele showed an increase of 18.1% in grain yield (Figure 2a), due to a significant increase in grains/spike (Figure 2b) and biomass (Figure 2c), compared with plants carrying the 2174 *TaNUE1* allele in the low‐N scenario. These field results further confirmed that *TaNUE1* in the targeted region affected grain yield in the low‐N scenario.

### Identification of candidate genes for *TaNUE1*

Among the three candidate genes, only *TaVRN‐A1* showed allelic variation in the promoter and introns, but there was no significant difference in gene expression between the two *TaVRN‐A1* alleles before N was used and within 2 weeks after N was used (Figure S3). The result suggested that the effects of *TaNUE1* on the mapped traits should rely on its protein sequence but not the transcript level.

**Figure 3:**
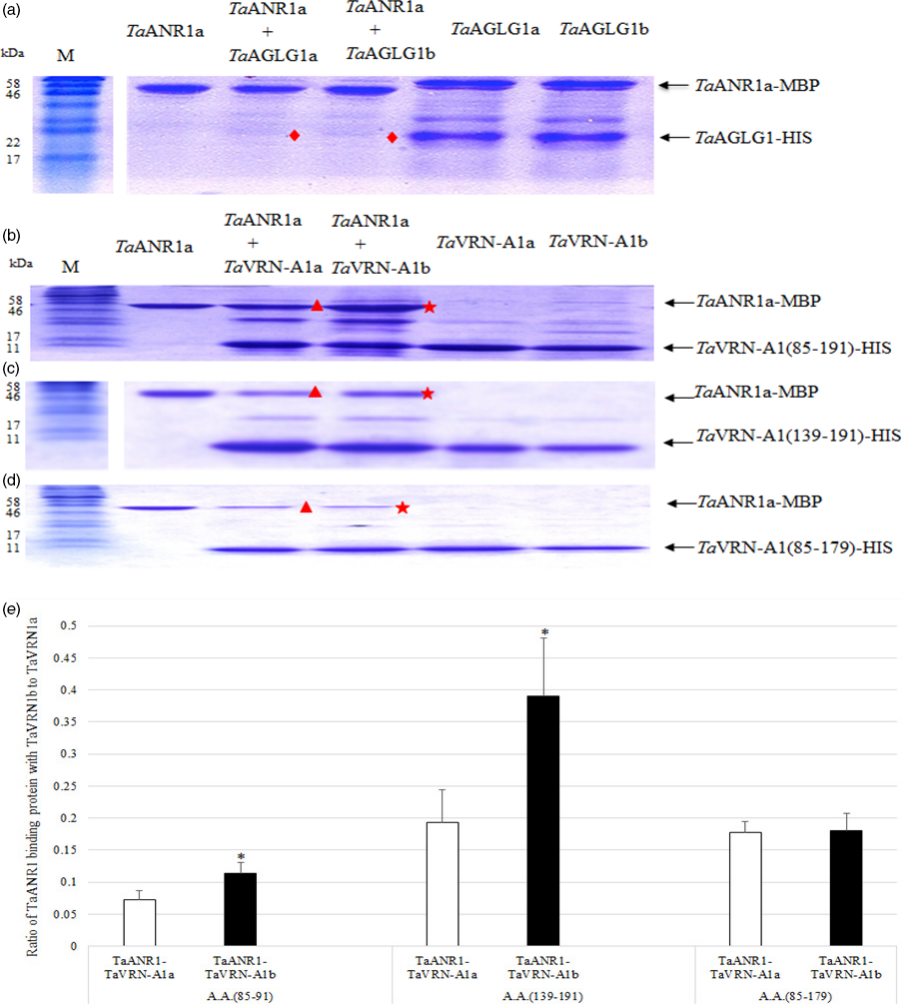
*In vitro* interactions between MADS proteins. *Ta* ANR1 is a protein with MBP‐tag, and *Ta* VRN‐A1 and *Ta* AGLG1 are the proteins with HIS‐tag. (a) The protein interaction of *Ta* ANR1a (60.1 kDa) and Jagger *Ta* AGLG1a protein (23.79 kDa) or 2174 *Ta* AGLGb (23.78 kDa). Diamonds represent an area where no interacting proteins are observed between the *Ta* ANR1a and *Ta* AGLG1 proteins. (b) The protein interaction of *Ta* ANR1a with *Ta* VRN‐A1 (85–191) including both Leu^117^/Phe^117^ and Ala^180^/Val^180^ substitutions. The interacting proteins of *Ta* ANR1a with the 2174 *Ta* VRN‐A1b (15.8 kDa) indicated by a star are stronger in protein band intensity than the interacting proteins of *Ta* ANR1a with the Jagger *Ta* VRN‐A1a (15.7 kDa) indicated by a triangle. (c) The protein interaction of *Ta* ANR1a with *Ta* VRN‐A1 (139–191) including Ala^180^/Val^180^ substitution only. The interacting proteins of *Ta* ANR1a with the 2174 *Ta* VRN‐A1b (9.3 kDa) indicated by a star are stronger in protein band intensity than the interacting proteins of *Ta* ANR1a with the Jagger *Ta* VRN‐A1a (9.3 kDa) indicated by a triangle. (d) The protein interaction of *Ta* ANR1a with *Ta* VRN‐A1 (85–179) including Leu^117^/Phe^117^ substitution only. The interacting proteins of *Ta* ANR1a with the 2174 *Ta* VRN‐A1b (14.5 kDa) indicated by a star are similar in intensity than the interacting proteins of *Ta* ANR1a with the Jagger *Ta* VRN‐A1a (14.4 kDa) indicated by a triangle. At least three independent replicates were performed for each of these interactions. M indicates a protein marker. (e) Comparison of interacted *Ta* ANR1 proteins between *Ta* VRN‐A1a and *Ta* VRN‐A1b proteins.

Among the three candidate proteins for *Ta*NUE1, there were no differences between the proteins encoded by the two alleles of *Ta*CYB5; this gene was thus excluded as a candidate. At the protein sequence, *Ta*AGLG1 had one amino acid substitution, whereas *Ta*VRN‐A1 had two. Next, we tested whether the two MADS proteins, *Ta*AGLG1 and *Ta*VRN‐A1, interacted with the wheat orthologue of ANR1 in Arabidopsis, which is also a MADS‐box protein. *ANR1* has a key role in regulating lateral root growth in response to changes in the external NO_3_^−^ supply (Zhang and Forde, 1998). *TaANR1* (GenBank Accession Number AM502900) had 70% similarity to the Arabidopsis ANR1 (GenBank Accession Number CAB09793). MADS‐box proteins may form a protein complex, in which one MADS‐box protein may positively or negatively regulate the expression of another MADS‐box gene via a direct interaction (Jack, 2004). *Ta*VRN‐A1, *Ta*AGLG1 and *Ta*ANR1a from Jagger were expressed in *Escherichia coli*. In pull‐down assays, no interaction was observed between *Ta*ANR1a and *Ta*AGLG1 (Figure 3a). However, the two *Ta*VRN‐A1 proteins encoded by the Jagger and 2174 alleles interacted differently with the*Ta*ANR1a protein (Figure 3b–e).

The alignment of *Ta*VRN‐A1 and *Ta*ANR1a proteins suggested that they might have interacting sites in the K box (Figure S4). The critical sites at which the Jagger *Ta*VRN‐A1a and 2174 *Ta*VRN‐A1b proteins interact with *Ta*ANR1a were identified by three experiments. *Ta*VRN‐A1a(85–191) and *Ta*VRN‐A1b(85–191), which included both Leu^117^/Phe^117^ and Ala^180^/Val^180^ substitutions, interacted differentially with *Ta*ANR1a (Figure 3b). Similarly, *Ta*VRN‐A1a(131–191) and *Ta*VRN‐A1b(139–191), which included an Ala^180^/Val^180^ substitution but not a Leu^117^/Phe^117^ substitution, interacted differentially with *Ta*ANR1a (Figure 3c). However, *Ta*VRN‐A1a(85–179) and *Ta*VRN‐A1b(85–179), which included a Leu^117^/Phe^117^ substitution but not an Ala^180^/Val^180^ substitution, interacted with *Ta*ANR1a in a similar manner (Figure 3d). The intensity of interacted *Ta*ANR1a proteins was significantly stronger in the pull‐down *Ta*VRN‐A1b protein than the pull‐down *Ta*VRN‐A1a protein including the Ala^180^/Val^180^ substitution (Figure 3e). These comparative *in vitro* interaction studies indicated that the differential interaction of *Ta*VRN‐A1a and *Ta*VRN‐A1b with *Ta*ANR1a was caused not by the Leu^117^/Phe^117^ substitution but by the Ala^180^/Val^180^ substitution.

**Figure 4:**
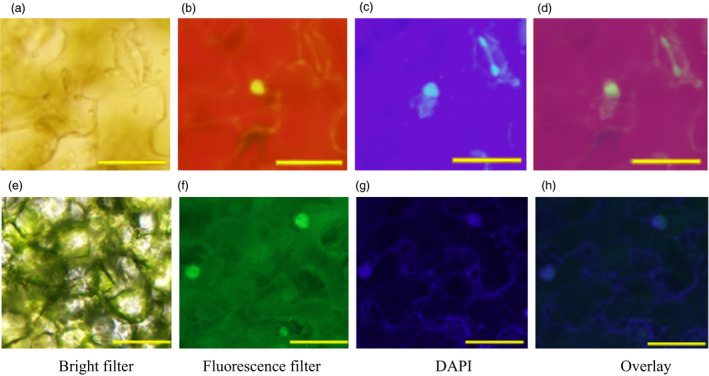
*In vivo* interactions of *Ta* VRN‐A1 with *Ta* ANR1 and *Ta* HOX1. (a–d) *In vivo* interaction between *Ta* VRN‐A1a‐pEG201‐YN and *Ta* ANR1a‐pEG202‐YC proteins, (e–h) *In vivo* interaction between *Ta* HOX1a‐YN and *Ta* ANR1a‐YC proteins. The paired proteins were simultaneously expressed in a living cell in *Nicotiana tabacum* (tobacco) leaves. (a) and (e) Image of the interacting proteins under a fluorescence microscope with a bright filter (BF). (b) and (f) Image of the interacting proteins under a fluorescence microscope with a green filter. (c) and (g) Image of the nucleus stained with 4′,6‐diamidino‐2‐phenylindole (DAPI). (d) and (h) Overlay images for the alignment of the interacting proteins with the DAPI‐stained nucleus. The scale bar in all images is 50 μm.

Next, *Ta*VRN‐A1 and *Ta*ANR1a were confirmed to interact with each other *in vivo* using bimolecular fluorescence complementation (BiFC). When *Ta*ANR1a was cloned into a pEG101‐YFP vector and the construct transformed into tobacco leaves, enriched yellow fluorescent signals of *Ta*ANR1a‐YFP were detected predominantly in the nucleus (Figure S5). The same pattern was observed for *Ta*VRN‐A1 (Li *et al*., 2013). When *Ta*VRN‐A1a was cloned into a pEG201‐YN vector, and *Ta*ANR1a was cloned into a pEG202‐YC vector, and these constructs were simultaneously expressed in the same cell, yellow fluorescence was again observed in the nucleus, confirming the *in vivo* interactions between these two proteins (Figure 4a–d). The differential interactions of *Ta*VRN‐A1a and *Ta*VRN‐A1b with *Ta*ANR1a provided initial evidence that *Ta*VRN‐A1 might be the better candidate for *Ta*NUE1.

### Regulation of *TaVRN‐A1* in normal and transgenic wheat plants

**Figure 5:**
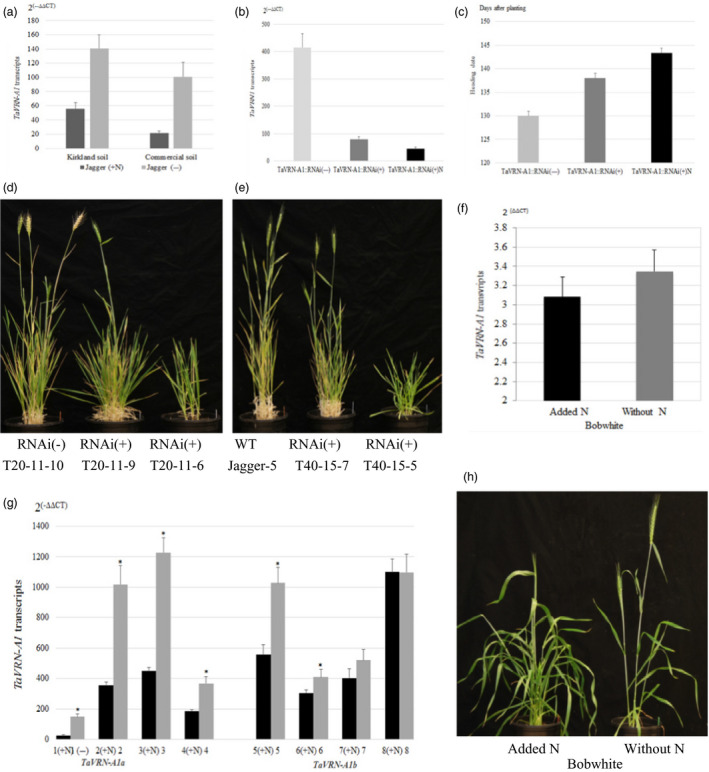
Regulation of *TaVRN‐A1* by N in normal and transgenic wheat plants. (a) Regulation of *TaVRN‐A1* transcript levels by N in Jagger grown in two different soils. (b) *TaVRN‐A1* transcript levels in *TaVRN‐A1::RNAi* transgenic Jagger wheat grown in the Kirkland soil. (c) Heading date of transgenic Jagger wheat grown in the Kirkland soil. *TaVRN‐A1::RNAi*(+) indicates positive transgenic plants, whereas *TaVRN‐A1::RNAi*(−) indicates nontransgenic plants. (d) Comparison of typical transgenic positive plants carrying *TaVRN‐A1::RNAi*(+), and nontransgenic plants of the T20 line at the heading stage. (e) Comparison of a typical transgenic plant carrying *TaANR1::RNAi* of the T40 line, and wild‐type Jagger plants at the heading stage. (f) Effects of N on *TaVRN‐A1* transcript level in the spring wheat cultivar Bobwhite. (g) Effects of N on *TaVRN‐A1* transcript level in eight vernalized winter wheat cultivars/lines. The dark columns indicate that N was utilized, whereas the grey columns indicate without N. 1. Jagger; 2. OK12716R/W; 3. OK11D25056; 4. Bentley, 5. Duster; 6. Gallagher; 7. Ruby Lee; 8. IBA; (+N) indicates that N was applied to the plants. Cultivars 1–4 carry the *TaVRN‐A1a* allele, whereas cultivars 5–8 carry the *TaVRN‐A1a* allele. (h) Effects of N on heading date in the spring wheat cultivar Bobwhite. Gene transcript levels were calculated using the 2^(−ΔΔ^ ^CT^ ^)^ method, where CT is the threshold cycle. Primers for *TaVRN‐A1* and *actin* used as an endogenous control are provided in Table S7. The values represent mean expression levels (n = 7–16), and the bar indicates standard error. Asterisk indicates that the difference was significant between the two alleles (*Pr* < 0.05).

A link between *TaVRN‐A1* and N regulation was discovered not only *via* its protein interaction with *Ta*ANR1, but also through its expression being regulated by N. In a separate experiment, four RILs carrying the Jagger *TaVRN‐A1a* allele were tested in the same Kirkland soil and the same commercial soil. When N fertilizer was applied to plants grown for 11 weeks without fertilizer, transcription of the *TaVRN‐A1a* allele was greatly down‐regulated after 3 weeks (Figure 5a).

Next, the RNA interference (RNAi) approach was used to disrupt *TaVRN‐A1a* expression in Jagger, and two individual transgenic plants (T20 and T40) were successfully generated. Transgenic T_1_ populations were tested in the same Kirkland soil used to identify *QNue.osu‐5A*. Transcription of *TaVRN‐A1a* was greatly reduced in the positive transgenic plants compared with nontransgenic plants (Figure 5b). Furthermore, the down‐regulatory effects of *TaVRN‐A1a* by RNAi were reflected in the heading date—for nontransgenic plants, this was 130 days, while it was 138 days for the positive transgenic plants (Figure 5c). In the positive plants, transcription of *TaVRN‐A1a* was further down‐regulated by N, compared with unfertilized positive plants (Figure 5b). After N fertilization, the heading date of positive transgenic plants was delayed to 143.4 days (Figure 5c). When T_2_ populations of T20 (Figure 5d) and T40 (Figure 5e) lines were tested in the commercial soil without vernalization, a typical positive plant carrying *TaANR1::RNAi* demonstrated delayed heading, fewer fertile tillers and decreased grain yield, compared with either the nontransgenic plants or the wild‐type Jagger.

Down‐regulation of *TaVRN‐A1* transcripts by N was also observed in six of seven winter wheat cultivars/lines that were naturally vernalized under field conditions. Vernalized plants were moved into a greenhouse where nutrients, temperature and light were controlled. Transcription of *TaVRN‐A1* significantly decreased 3 weeks after application of N (Figure 5g). Down‐regulation of *TaVRN‐A1* (Figure 5f) and delayed heading and increased biomass by N (Figure 5h) were also observed in spring wheat *cv*. Bobwhite.

### Function of *TaANR1* in a natural mutant and transgenic wheat

**Figure 6:**
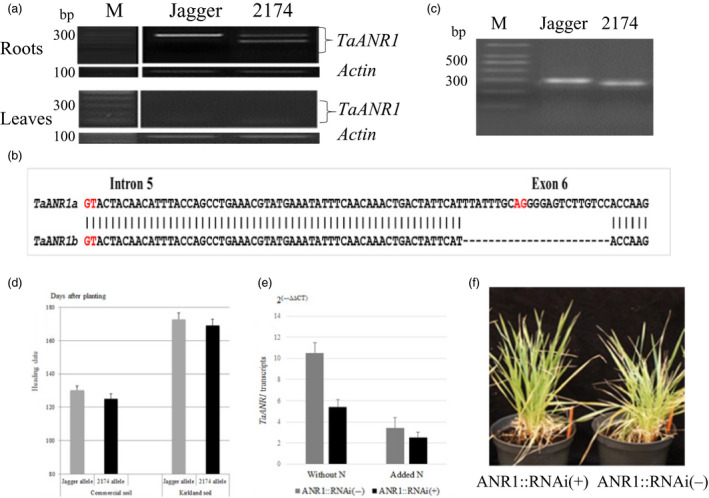
Function of *TaANR1* in natural mutant and transgenic plants. (a) Comparison of *TaANR1* expression patterns in root and leaf samples between Jagger and 2174. Primers ANR‐MS‐F2 (5′‐GAATGGGTGTCAAGGAACTGCAGG‐3′) and ANR‐MS‐R2 (5′‐GGAGTTCTTGAATTTCGGTTAACTTCAGTCA‐3′) were designed to amplify the 250‐bp Jagger allele and the 166‐bp 2174 allele. (b) A diagram of 23‐bp indel in locations and sequences of *TaANR1* between the Jagger and 2174 alleles. The splicing sites at 5′ end (GT) and 3′ end (AG) of intron 5 are highlighted in red. (c) A PCR marker for 23‐bp indel between the Jagger *TaANR1a* allele and the 2714 *TaANR1b* allele. Primers ANR‐MF1 (5′‐ATCACAAGGTACTACAACATTTAC‐3′) and ANR‐MR1 (5′‐GGAGTTCTTGAATTTCGGTTAACTTCAGTCA‐3′) were designed to amplify the Jagger allele (286 bp) and the 2174 allele (263 bp). M: DNA marker. (d) Genetic effect of *TaANR1* on heading date of RILs in the commercial soil and Kirkland soil. (e) Regulation of *TaANR1a* transcripts by N and RNAi transgenic wheat. Transcript levels were determined using values calculated by the 2^(−ΔΔ^ ^CT^ ^)^ method, where CT is the threshold cycle. The values represent mean expression levels (n = 8), and the bars indicate standard errors. (f) Comparison of a typical transgenic plant carrying *TaANR1::RNAi* and nontransgenic plant at the juvenile stage.

Two pieces of evidence confirmed that the *TaANR1* orthologue is also regulated by N in wheat. Firstly, a natural mutant of *TaANR1* was found to have a genetic effect on wheat development and growth. When conserved primers for homoeologous *TaANR1* genes were designed to test expression profiles, *TaANR1* transcripts were found not visibly in leaves but predominantly in roots in both Jagger and 2174. The PCR product was 250 bp in size in Jagger, but showed an additional band besides the 250‐bp band in 2174 (Figure 6a). Sequence analysis indicated that the cDNA products with the single band from Jagger were a mixture of homoeologous *TaANR1* transcripts from chromosome 2A (TGACv1_scaffold_093384_2AL) and chromosome 2D (TGACv1_scaffold_159992_2DL). In 2174, the cDNA products of the upper band were from *TaANR1* on chromosome 2D, and the cDNA products of the lower band were from *TaANR1b* on chromosome 2A; however, the cDNA fragment of the *TaANR1b* gene was 166 bp in size because the 84‐bp exon 6 was missing. To determine whether this missing exon 6 was caused by an exon‐skipping event, or by a deletion event at the gDNA level, the gDNA products of this gene were cloned and sequenced. Results showed that the lack of exon 6 in its cDNA was caused by a 23‐bp deletion event comprising 10‐bp at the 5ʹ end of intron 5 and 13‐bp of exon 6 in the gDNA sequence of 2174 (Figure 6b). This included the AG splice site at the 5ʹ end of intron 5, resulting in the loss of the full 84‐bp exon 6 in its mRNA, and thus 28 amino acids. These missing amino acids were included in the interaction site of *Ta*ANR1 with *Ta*VRN‐A1 (Figure S4).

Based on the 23‐bp indel, a polymorphic PCR marker for *TaANR1* was developed (Figure 6c). When phenotypic data of the RIL population used to map *QNue.osu‐5A* were analysed for genetic effects on *TaANR1*, there was a difference of 5.2 days in plants grown in commercial soil and 3.9 days in plants grown in Kirkland soil between the two alleles (Figure 6d). This result indicated that the Jagger *TaANR1a* gene is a minor heading repressor in wheat. The effect of *TaANR1* on grain yield in the RIL population tested in the field was described below. The natural *TaANR1b* mutant allele exists in 13 of 69 wheat cultivars across the USA, but 12 of the 13 *TaANR1b* wheat cultivars were utilized in the Southern Great Plains (Table S5).

Secondly, three individual transgenic Jagger plants with *TaANR1::RNAi* were successfully generated, and their T_1_ populations were tested in the commercial soil. *TaANR1* transcription was down‐regulated in positive plants compared with nontransgenic plants, and *TaANR1* transcription was down‐regulated by N in the positive transgenic plants and nontransgenic plants (Figure 6e). Compared with nontransgenic plants, the heading date of transgenic plants was 2.5 days earlier—this confirms that *TaANR1* has a minor and repressive effect on heading. A typical positive plant carrying *TaANR1::RNAi* showed an earlier developmental stage but fewer tillers during the juvenile phase compared with a nontransgenic plant (Figure 6f).

### *Ta*ANR1 and *Ta*HOX1 bound to the Ala^180^/Val^180^ position of *Ta*VRN‐A1

The Ala^180^ in the *Ta*VRN‐A1a protein was found to be critical for maintaining the ability to interact with *Ta*HOX1, a homeobox protein involved in wheat heading date, and the mutated Val^180^ in *Ta*VRN‐A1b decreased the ability to bind with *Ta*HOX1 (Li *et al*., 2013). In this study, the Ala^180^ in the *Ta*VRN‐A1a protein was less able to interact with *Ta*ANR1a than the mutated Val^180^ in *Ta*VRN‐A1b (Figure 3b–d). These results suggest that *Ta*VRN‐A1, *Ta*ANR1 and *Ta*HOX1 might form a protein complex in wheat. To test whether *Ta*ANR1 interacts with *Ta*HOX1, these two proteins were analysed for *in vitro* and *in vivo* interactions.

No significant interaction was detected between *Ta*ANR1a and *Ta*HOX1 *in vitro* (Figure S6). However, *Ta*ANR1a interacted with *Ta*HOX1 *in vivo* (Figure 4e–h). This indicated that *Ta*ANR1a and *Ta*HOX1 bound to the Ala^180^/Val^180^ position of *Ta*VRN‐A1. The Jagger *Ta*HOX1a protein has a leucine residue at position 99 that is substituted with a proline residue in the 2174 *Ta*HOX1b protein. Both *Ta*HOX1a and *Ta*HOX1b showed *in vivo* interactions with *Ta*ANR1a. These results suggest that the differential interactions between *Ta*VRN‐A1, *Ta*ANR1 and *Ta*HOX1 proteins might modify N‐related traits.

### Genetic effects of *TaVRN‐A1*,* TaANR1* and *TaHOX1* on grain yield in the field

To evaluate the combined effects of favourable alleles of *TaVRN‐A1*,* TaANR1* and *TaHOX1* on grain yield, we tested the effects of natural mutations on grain yield in the 96 RIL population under field conditions for 2 years and in two locations. This approach circumvented regulatory roadblocks associated with cultivating genetically modified crops.

**Figure 7:**
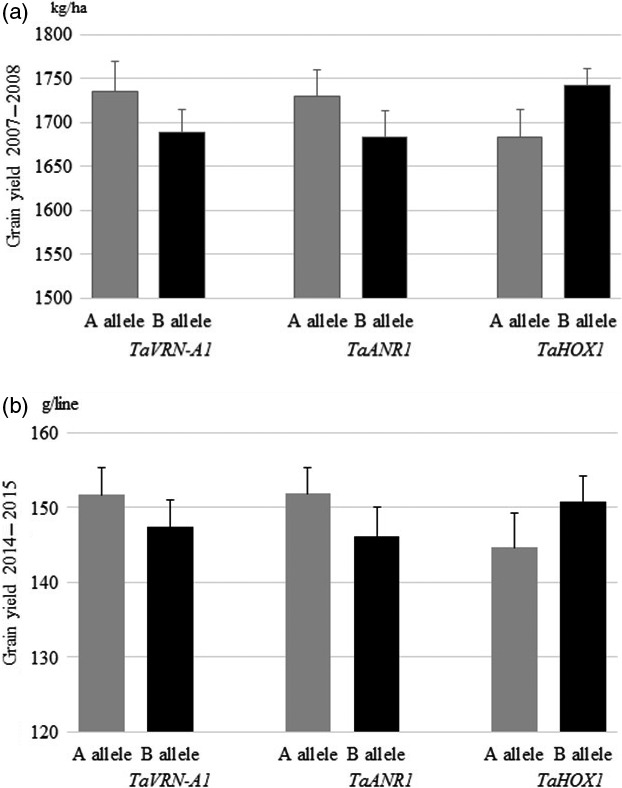
Genetic effects of *TaVRN‐A1*,* TaANR1* and *TaHOX1* on grain yield in the field. (a) Genetic effect of three genes on grain yield in recombinant inbred lines (RILs) tested in the field in the 2007–2008 growing season. (b) Genetic effect of three genes on grain yield in RILs tested in the field in the 2014–2015 growing season. The average values of each gene in the population of 96 Jagger × 2174 RILs were compared for two alleles: ‘A’ for those lines (n = 25–58) carrying the Jagger allele, and ‘B’ for those lines (n = 48–61) carrying the 2174 allele. The bars indicate standard errors.

When tested in the field at high N levels in the 2007–2008 growing season, lines carrying the Jagger *TaVRN‐A1* allele achieved a higher grain yield by an average of 2.82% compared with plants carrying the 2174 *TaVRN‐A1* allele (Figure 7a). The Jagger *TaVRN‐A1* allele increased grain yield by 2.92% in the 2014–2015 growing season, when a population of the same RIL was grown in a field with sufficient N to attain high grain yield (Figure 7b). The *TaANR1a* in Jagger was observed to increase grain yield. Lines carrying the Jagger allele achieved higher grain yields by an average of 3.39% (2.8% in the 2007–2008 growing season, and 3.97% in the 2014–2015 growing season), compared with plants carrying the 2174 allele (Figure 7a, b). *Ta*HOX1a had a repressive effect on grain yield. Lines carrying the Jagger *TaHOX1a* allele achieved a lower grain yield by a decrease of 4.46% from 4.69% on average in the 2007–2008 growing, and 4.22% in the 2014–2015 growing season compared with plants carrying the 2174 *TaHOX1b* allele (Figure 7a, b). Together, *TaVRN‐A1a* and *TaANR1a* in Jagger and *TaHOX1b* in 2174 significantly increased grain yield by an average of 10.71% (9.84–11.58%) (*Pr* < 0.05), based on the average grain yield of the 96 RILs tested in the field for 2 years and in two locations.

## Discussion

In this study, we used two contrasting N fertilization regimes to map *QNue.osu‐5A* and identified *TaVRN‐A1* as *TaNUE1* in winter wheat. In previous studies, *TaVRN‐A1* was cloned as a gene for variations in the qualitative vernalization requirement between spring wheat and winter wheat (Yan *et al*., 2003) and for the quantitative vernalization requirement between weak winter wheat and strong winter wheat using a positional cloning approach (Li *et al*., 2013). The *TaVRN‐A1* locus on chromosome 5AL was also mapped and directly linked to key wheat and barley traits such as frost tolerance, spike architecture and plant height, tiller and spikelet number, leaf length, grain yield, nitrogen uptake and NUE (An *et al*., 2006; Deng *et al*., 2015; Fowler and Gusta, 1977; Habash *et al*., 2007; Hayes *et al*., 1993; Kato *et al*., 2000; Quraishi *et al*., 2011; Roberts, 1990; Snape *et al*., 1985). In addition to the *Vrn‐A1* locus, the *Vrn‐D1* locus was associated with QTL for traits related to NUE components in wheat in the same population of the Chinese Spring × SQ1 population (Habash *et al*., 2007). However, the genes in the mapped regions that controlled NUE and the related agronomic traits were unknown.

Results from the present study indicated that it is *TaVRN‐A1* that is regulated by N and linked to the multiple agronomic traits. The conclusion was drawn based on three pieces of experimental evidence. Firstly, the segregated NUE and related agronomic traits were associated with three candidates at the *TaVRN‐A1* locus, and the *TaVRN‐A1* gene was the best candidate responsible for the traits. Secondly, the Ala^180^/Val^180^ substitution in the *Ta*VRN‐A1 protein caused differential interactions with *Ta*ANR1, establishing a connection between the vernalization gene and the N‐regulated gene. The function of *TaANR1* in wheat was validated by the down‐regulation of *TaANR1* transcripts by N, and the delayed effect of *TaANR1* on heading date by RNA interference in transgenic plants and by analysis of a natural mutant. Lastly, RNA interference and N showed similar effects in the transgenic wheat in delaying heading date. However, precise effects of disrupted *TaANR1* expression on NUE need to evaluate in a field experiment.

Three homoeologous genes, *TaVRN‐A1*,* TaVRN‐B1* and *TaVRN‐D1*, exist in common wheat. In this study, only allelic variation in *TaVRN‐A1* was found to link with the segregated NUE and related agronomic traits in the winter wheat population, and only *Ta*VRN‐A1 was tested for its interaction with *Ta*ANR1. The homoeologous *TaVRN‐B1* and *TaVRN‐D1* and their proteins should also have similar effects on the N‐related agronomic traits, although the role of the genes in the N‐related agronomic traits under different genetic backgrounds needs to be investigated. In cereal crops, NUE refers to the ratio of grain yield to N supplied by soil and fertilizer, which is dissected into two components: N uptake (NU) and N utilization efficiency (UTE) (An *et al*., 2006; Moll *et al*., 1982). In this study, we used grain yield to describe NUE for two alleles for each of the three genes tested at the same N level. The allelic effect of *TaVRN‐A1a* on grain yield was increased to up to 140% when the population was grown in the Kirkland soil with limited N and in the greenhouse without vernalization. The allelic effect of *TaVRN‐A1a* on grain yield was increased to 18.1% when the critical recombinant lines with limited genetic backgrounds were grown under low‐N conditions in the field with natural vernalization. The enlarged effects observed in plants grown under low‐N conditions facilitated positional cloning of *TaNUE1*. In addition, the cloned *TaVRN‐A1* allowed for identification of its interactors in the same pathway. The results from this study suggest that the differential interactions between *Ta*VRN‐A1, *Ta*ANR1 and *Ta*HOX1 proteins might modify N‐related traits. It is a critical finding that *Ta*ANR1 and *Ta*HOX1 competed to bind to the Ala^180^/Val^180^ position of *Ta*VRN1. The competition between *Ta*ANR1 and *Ta*HOX1 for *Ta*VRN‐A1 is reflected in their effects on grain yield. As a competitor with *Ta*ANR1 for *Ta*VRN‐A1, *Ta*HOX1a had a repressive effect on grain yield. These genes caused subtle modifications to grain yield in a common high‐N scenario in the field, but genetically incorporating favourable alleles from the *TaVRN‐A1a* and *TaANR1a* genes in Jagger, and the *TaHOX1b* gene in 2174, increased grain yield by an average of 10.71% (9.84% to 11.58%). Favourable alleles of these genes involved in regulating nitrogen pathways can be pyramided to improve NUE and grain yield in novel wheat cultivars.

Nitrogen has paradoxical effects on development. Lines started from seed and grown in the commercial soil showed accelerated plant development compared with those grown in the Kirkland soil. However, when N was supplied to the plants prior to stem elongation, the plant heading date was delayed. Plants accelerate the transition to the reproductive phase and flower faster with lower nutrients (Martinez‐Zapater *et al*., 1994). This study indicated that when N was used to fertilize a plant prior to stem elongation, *TaVRN‐A1* transcripts were down‐regulated, and the plant heading date was thus delayed. This explains the molecular mechanism underlying the observation that plants flower faster in N‐deficient conditions. In winter wheat, *TaVRN‐A1* is induced by vernalization, light or plant age (Danyluk *et al*., 2003; Li *et al*., 2013; Murai *et al*., 2003; Trevaskis *et al*., 2003; Yan *et al*., 2003). Without vernalization, *TaVRN‐A1* is expressed in spring wheat because of the presence of multiple mutation forms, including indels of different forms in the promoter and the first intron (Dubcovsky *et al*., 2006; Fu *et al*., 2005; Pidal *et al*., 2009; Yan *et al*., 2003, 2004), a retrotransposon insertion in the 5ʹ UTR (Chu *et al*., 2011), an miRNA binding site in a miniature inverted‐repeat transposable element in the promoter (Yu *et al*., 2014) or the binding site of *TaGRP2* in intron 1 (Kippes *et al*., 2015). This study demonstrated that N regulates *TaVRN‐A1* and flowering in both spring wheat and winter wheat cultivars.

## Materials and methods

### Plant materials and experimental soils

#### Mapping *QNue.osu‐5A in RILs in low‐N soil*

The population of RILs was generated from a cross between two locally adapted winter wheat cultivars, ‘Jagger’ and ‘2174’ (Chen *et al*., 2009). A population of 96 RILs was used to initially map traits related to NUE. A Kirkland silt loam soil was collected from the Cimarron Valley Research Station, OK (USA); the soil was low in N, but had normal levels of other essential nutrients (Table S1). The soil was thoroughly mixed using a cement mixer before being distributed equally into pots. Three plants of a single RIL were grown in each pot (10 cm in diameter and 12 cm in height) with 1.8 kg soil in a greenhouse in Stillwater, OK. The greenhouse was maintained at a constant temperature of 25/20 °C day/night with long days (16 h light/8 h darkness), and additional sodium lamps were supplied. The winter wheat population was not subjected to vernalization, to avoid interactive effects between low temperatures and the traits of interest.

Leaf greenness and total N of aboveground biomass were assessed from one of the replicates. Heading date was scored when a single plant had completely emerged from the boot. Leaf chlorophyll content was assayed using a SPAD 502 Chlorophyll Meter (Konica Minolta Sensing, Inc., Osaka, Japan). The dry weight of the shoot tissues in mature plants was weighed to determine biomass. Harvest index was the ratio of total grain weight to total aboveground biomass. Tiller number was counted from ten plants at the end of the stem elongation stage when tillers reached their maximum number. Spike number was counted at maturity, and the total number of grains from a plant was counted to calculate the number of grains per spike. Grains were dried at 65 °C for 2 days; grain weight was the weight of the dried grain plus 13% standard moisture per thousand grains (thousand kernel weight). These yield components were phenotyped from three plants tested in the Kirkland soil in the greenhouse.

PCR markers for the allelic variation of each gene were developed and mapped in the RILs (Figure S2). Markers for three candidate genes were developed based on the sequences deposited in GenBank, including JQ915055 for Jagger and JQ915056 for 2174 of *TaVRN‐A1*, JQ915057 for Jagger and JQ915058 for 2174 of *TaAGLG1*, JQ915059 for Jagger and JQ915060 for 2174 of *TaCYB5*.

#### QTL validation and statistical analysis

*QNue.osu‐5A* was identified in the same population of recombinant inbred lines (RILs) grown under two contrasting N fertilization regimes in the same greenhouse. The genetic linkage group utilized to identify genes for stem elongation time (Chen *et al*., 2009) and vernalization requirement duration (Li *et al*., 2013) was used to discover *QNue.osu‐5A*. WinQTLCart 2.5 (North Carolina State University, Raleigh) (Wang *et al*., 2007) was used to conduct analyses for the N‐related traits using interval mapping (IM). A QTL was declared when the logarithm of the odds (LOD) score exceeded the threshold value of 2.5, and the significance level was at 0.05. LOD values and genetic partitioning of the total phenotypic variation are presented as generated from the QTL program, using standard procedures. One‐way analysis of variance (ANOVA) was used to analyse the interactions of the QTLs with N rate for each of the 10 traits using the SAS procedure CORR (SAS Institute, Cary, NC). A QTL was statistically claimed to be regulated by N, when the genotype x nitrogen environment effect was significant.

The mean value of each trait from plants carrying the same allele was used for ANOVA. The *t*‐test was used to determine the significance level to compare mean differences between treatments. Correlation analysis examined the association of two phenotypes in the same treatment.

#### Testing critical recombinant lines

Fourteen recombinant BC_1_F_2_ and three recombinant BC_1_F_3_ lines (Figure 1c) were tested with the same Kirkland soil. Sixty plants of each recombinant line were tested under the same greenhouse conditions used to discover *QNue.osu‐5A*. A commercial potting mix, Sunshine Redi‐earth growing mix (Sungro Horticulture Canada Led.) was used in the experiments, which includes fine Canadian sphagnum peat moss, vermiculite and dolomitic limestone, and has a nutrient system delivering a supply of N for 6 weeks. Whenever appropriate, N from Miracle‐Gro^®^ Water Soluble All Purpose Plant Food or urea fertilizer was dissolved in water and poured into pots. The heading date was scored when a single plant head had completely emerged from the boot.

At the Cimarron Valley Research Station in the 2011–2012 growing season, seven critical recombinant lines were grown in a Teller soil with 7.5 ppm NO^3−^N, representing a N‐stress condition (Table S1). A forage crop was grown for two consecutive seasons, and no N was supplied to both lower and level the available N in the Teller soil. The experimental design was a randomized complete block with three replications. Yield components were phenotyped from 10 plants per line tested in the Teller soil in the field.

#### Testing the RIL population in the field

Yield experiments on 96 RILs of the Jagger × 2174 population were conducted for 2 years. The first experiment took place at the North Central Agronomy Research Station near Lahoma, OK, and the population was planted as a standard yield plot on October 30, 2007. The plot area (3 m × 1.26 m) was fertilized before planting according to soil‐test recommendations for adequate N to attain a yield goal of approximately 3000–6700 kg/ha in this specific site (Edwards *et al*., 2012). The second experiment was conducted at the Agronomy Research Station in Stillwater, OK, and the population was planted as a head‐row plot on 14 November 2014. At the experiment site, the top 30 cm of soil contained 40 ppm N, 16 ppm P_2_O_5_ and adequate K with a pH of 5.3. Diammonium phosphate (DAP, 8‐5‐14) fertilizer of 309 kg/ha and urea of 371 kg/ha were incorporated into the soil by cultivator tillage. The experimental design was a randomized complete block with two replications. Dry grains for each plot were weighed to determine grain yield.

### Regulation of *TaVRN‐A1* by N in different cultivars/lines

Three cultivars/lines carrying the *TaVRN‐A1a* allele (Bentley, OK12716R/W, and OK11D25056) and four cultivars carrying the *TaVRN‐A1b* allele (Duster, Gallagher, IBA, and Ruby Lee) were first planted in the field for an annual variety trial at Stillwater on 14 October 2015. Plots were 3.0 m in length, with 30 cm between rows. At the experiment site, the top 30 cm of soil contained 47 ppm N, 39 ppm P_2_O_5_ and 66 ppm K_2_O, with a pH of 5.3. Diammonium phosphate (DAP, 18‐46‐0) fertilizer of 56 kg/ha was also supplied in‐furrow. On 24 February 2016, 130 days after planting, six plants of each cultivar, which were expected to have satisfied their vernalization requirement in the natural field condition, were transferred to pots with commercial soil in the greenhouse and N fertilizer at the 100 kg N ha^−1^ level was supplied. Three weeks after fertilization, leaf samples from three plants were collected to test the regulation of gene expression by N. Unfertilized plants were used as controls.

### *In vitro* protein interactions

The three cDNAs of *TaVRN‐A1a* and *TaVRN‐A1b* encoding proteins from 85–191 aa, 85–179 aa, and 139–191 aa; one cDNA of *AGLG1a* and *AGLG1b* encoding proteins from 1 to 180 aa; and one cDNA of *TaHOX1a* and *TaHOX1b* encoding proteins from 1 to 150 aa, were each cloned into a pSKB3 vector with an N‐terminal 6×HIS‐tag (Li *et al*., 2013). The cDNAs encoding proteins from 111 to 240 aa (end) for *Ta*ANR1a, and from 111 to the end for *Ta*ANR1b, were cloned into a pMAL‐c2X vector with an MBP‐tag (New England BioLabs, Ipswich, MA, USA). The primers used for cloning are listed in Table S6. Constructs were expressed in *E. coli* (BL21 DE3). An Ni‐NTA column (Qiagen, Germantown, MD, USA) was used to purify the expressed proteins from pSKB3 with the 6×HIS‐tag, and an amylose column (New England BioLabs) was used to dialyse and purify the protein fused with the MBP‐tag.

The protein fused with the 6×HIS‐tag (500 μg/mL, 50 μL) was incubated in an Ni‐NTA column (40 μL) for 1 h at 4 °C in binding buffer with 10 mm imidazole, and the MBP‐tag protein (500 μg/mL, 100 μL) was then added to the column. MBP‐tag protein (500 μg/mL, 100 μL) alone was added to an Ni‐NTA column as a negative control for nonspecific interactions between MBP‐tag and the Ni‐NTA column. Reactions were incubated overnight at 4 °C. The interacting proteins in the resin were then washed with chilled binding buffer with 35 mm imidazole six or eight times until the MBP‐tag protein disappeared in the negative control. The protein was eluted by boiling the samples in 40 μL of sodium dodecyl sulphate polyacrylamide gel electrophoresis (SDS‐PAGE) loading buffer for 5 min. The eluted protein samples (5 μL) were analysed on a 12% SDS‐PAGE gel stained with Coomassie Brilliant Blue. When the protein fused with the MBP‐tag was incubated in the amylose column, the 6×HIS‐tag protein was added to the amylose column and then washed with column buffer until the 6×HIS‐tag protein disappeared in the negative control. Interacting proteins were analysed in pull‐down assays.

ImageJ 1.32 software (National Institutions of Health, Bethesda, MD. http://rsb.info.nih.gov/ij) was used to quantify intensity of interacting proteins. The amount of interacting proteins was converted to the percentage of its own intensity over the proteins collected from the same reaction.

### Subcellular localization and *in vivo* protein interactions

The complete cDNA of *TaANR1a* and the cDNA fragment of *TaHOX1a* encoding its protein from 1 to 150 aa were cloned into pDONR207 with the BP Cloning Kit (Invitrogen) according to the manufacturer's instructions. The LR Cloning Kit (Invitrogen, Waltham, MA, US) was then used to transfer *TaANR*1 to pEarleygate 101 (pEG101) for subcellular localization of *Ta*ANR1a. Primers used for constructs are provided in Table S6.

Previous reports located *Ta*VRN‐A1 and *Ta*HOX1 in the nucleus of the cell (Li *et al*., 2013). The *TaANR1* in pDONR207 was fused to the C‐terminal amino acid portion (175–239 aa) of YFP in the pEarleyGate202‐YC vector (pEG202‐YC) to test *in vivo* interactions with *Ta*VRN‐A1 (1–244 aa, end) or *Ta*HOX1 (1–150 aa), which was fused to the N‐terminal 174 amino acid portion (1–174 aa) of YFP in the pEarleyGate201‐YN vector (pEG201‐YN). Reciprocal empty vectors were also used as negative controls for interactions with *Ta*ANR1, *Ta*VRN‐A1 or *Ta*HOX1 proteins. *Agrobacterium tumefaciens* strains (GV3101) carrying the BiFC constructs and p19 strain were used together to infiltrate *Nicotiana benthamiana* leaves 5 weeks after planting. Leaves were ready for BiFC imaging after 3 days of infiltration. Images were visualized under a BX‐51 fluorescence microscope (Olympus). Images were taken with a bright filter (BF) to indicate the background of the leaves infiltrated with *A. tumefaciens* carrying constructs, or with an ultraviolet filter to indicate the position of the nucleus stained with 4′, 6‐diamidino‐2‐phenylindole (DAPI), and with GFP and GFPA filters to indicate the presence of fluorescent proteins. Scale bars in all images are 50 μm.

### Transgenic wheat

Two transgenic plants (T20 and T40) were generated using the *TaVRN‐A1::RNAi* constructs, and three transgenic plants (T1, T13 and T14) were generated using the *TaANR1::RNAi* construct. The primers used to amplify the genes are provided in Table S6. Amplified genes were inserted at the enzyme digestion sites (*Asc* I/*Avr* II, sense fragment; *Spe* I/*Asis* I, antisense fragment) of the RNAi vector pMCG161—this contains the BAR gene, which confers resistance to bialaphos. Constructs were transformed into the Jagger cultivar by particle bombardment using a published protocol (Sparks and Jones, 2014).

Transgenic wheat plants were tested in the same Kirkland soil used to discover *QNue‐osu‐5A*. The T_1_ of three lines of *TaANR1::RNAi* were tested in the commercial soil. All transgenic lines were tested under the same greenhouse conditions used to discover *QNue‐osu.5A* and without vernalization. Leaf samples were collected from *TaVRN‐A1::RNAi* lines 77 days after planting and before N fertilizer at the 100 kg N ha^−1^ level was applied to positive and negative plants. Leaf samples with/without N were collected for RNA extraction 3 weeks after fertilization. Root samples of *TaANR1::RNAi* lines were collected 56 days after planting and before N fertilizer at the 100 kg N ha^−1^ level was applied. Root samples for RNA extraction were collected 1 and 2 weeks after fertilization. Four T_1_ lines of T20 and T40 were selected to generate a T_2_ population for phenotyping in the commercial soil.

### Quantitative RT‐PCR

Total RNA from shoots and roots was extracted using TRIzol^®^ reagent (Invitrogen) according to the manufacturer's instructions. Root RNA was extracted using a modified method in the RNA precipitation step, whereby 0.25 mL of isopropanol was added, followed by 0.25 mL high salt precipitation solution (0.8 m sodium citrate and 1.2 m NaCl) per 1 mL TRIzol^®^ reagent. One microgram of RNA was treated with deoxyribonuclease I (Invitrogen), and complementary DNA (cDNA) was synthesized using a SuperScript™ II Reverse Transcriptase Kit (Invitrogen) with an oligo(dT)_20_ primer. Quantitative reverse transcription polymerase chain reaction (qRT‐PCR) was carried out using a 7500 Real‐time PCR System (Applied Biosystems, Foster City, CA, USA) and iQ™ SYBR^®^ Green Supermix (Bio‐Rad, Hercules, CA, USA) with a three‐step cycling program consisting of an initial denaturation step at 95 °C for 3 min, followed by 39 cycles at 95 °C for 15 s, 57 °C for 30 s and 72 °C for 30 s. Primers used in RT‐qPCR to amplify *TaVRN‐A1*,* TaANR1* and *actin* are listed in Table S7. Gene transcript levels were described using the values calculated by the 2^(−ΔΔCT)^ method (Li *et al*., 2013), where CT is the threshold cycle. All samples were subjected to two technical replicates and at least six biological replicates.

## Conflict of interest

The authors declare no conflict of interest.

## Supporting information


**Figure S1** Winter wheat plants in N‐deficient soil before and after fertilization.
**Figure S2** Markers for genes encompassing *TaNUE1*.
**Figure S3** Comparison of *TaVRN‐A1* expression level in Jagger versus 2174.
**Figure S4** Interacting site of *Ta*VRN‐A1 and *Ta*ANR1a proteins.
**Figure S5** The subcellular location *Ta*ANR1‐YFP protein in living cells of tobacco leaves.
**Figure S6** *In vitro* interaction of *Ta*HOX1 and *Ta*ANR1a proteins.

**Table S1** Characteristics of the soils used for NUE.
**Table S2** LOD and variation of *QNue.osu‐5A* on 10 traits related to N utilization.
**Table S3** Regulatory effects of *QNue.osu‐5A* by N.
**Table S4** Wheat heading date of seven critical recombinant lines and parental lines tested in different soils.
**Table S5** Wheat cultivars used for determining the frequency of *TaANR1* alleles.
**Table S6** Primers for protein–protein and protein–DNA interactions, and gene transformation.
**Table S7** Primers for gene expression.
