# Supplementary material for: Nitrogen use efficiency is regulated by interacting proteins relevant to development in wheat
Source: Plant Biotechnol J. 2018 Jan 15;16(6):1214–26. doi: 10.1111/pbi.12864 (PMC5978868; doi:10.1111/pbi.12864)
Supplement: Supplementary file 1 — Figure S1 Winter wheat plants in N‐deficient soil before and after fertilization. Figure S2 Markers for genes encompassing TaNUE1. Figure S3 Comparison of TaVRN‐A1 expression level in Jagger versus 2174. Figure S4 Interacting site of TaVRN‐A1 and TaANR1a proteins. Figure S5 The subcellular location TaANR1‐YFP protein in living cells of tobacco leaves. Figure S6 In vitro interaction of TaHOX1 and TaANR1a proteins. [file PBI-16-1214-s002.docx]

**Figure S1**

(c)


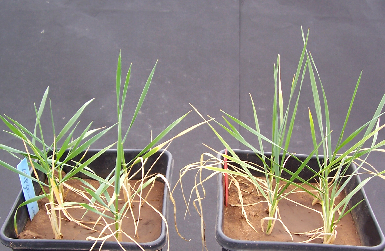

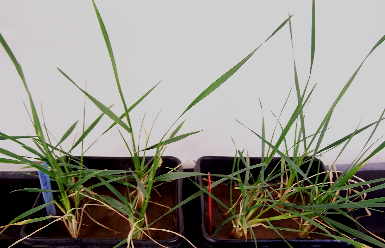

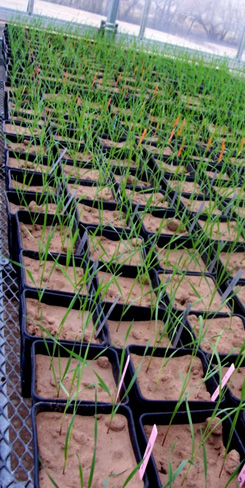


(a)

2174

Jagger

(b)

Jagger

2174

**Figure S1 Winter wheat plants in N-deficient soil before and after fertilization.**

**(a)** Plants were grown in Kirkland soil that was N deficient and normal for other nutrients. The experiments were conducted in a greenhouse, where temperature, photoperiod and moisture conditions were controlled in order to reduce interactions between genetic and environmental factors on N response. **(b)** The plants showed similar phenotypes at 11 weeks after planting. **(c)** The plants showed visible segregation in agronomic and physiological traits three weeks after fertilized with 54.06 mg N kg^-1^ soil (equivalent to 100 kg N ha^-1^).

**Figure S2. Marker for genes encompassing *TaNUE1.***

**Marker for *GT***

Primers for mapping of the *GT* gene are GT-F6 (5’-CAGGTACGTGACAGAGATCGA-3’) and GSGT-R3 (5’-CCTCTGCCAATCCAGACGATGG-3’)*.* The primers amplified 789 bp fragment by using regular PCR at 55ºC for annealing temperature and 1 min for extension. The PCR products were digested with restriction enzyme *Rsa* I, and he polymorphic bands were 257 bp for the Jagger allele and 224 bp for the 2174 allele.


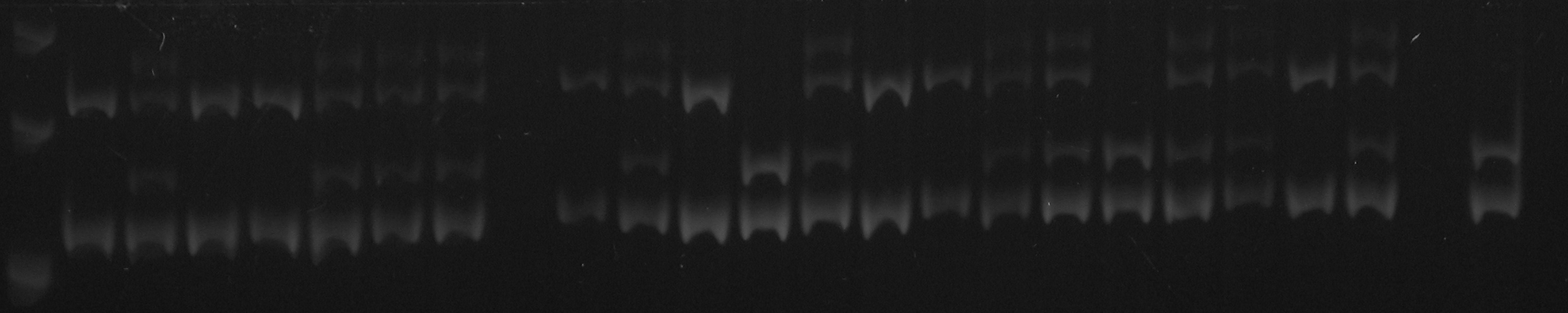


Jagger Het. Het. 2174

**Marker for *STR***

Primers for the *STR* gene are STR-C2F (5’-ATCGTGTAGAGGTGCAGCAACTA-3’and STR-R3 (5’-TGAAAGATCCCAGGGACAAACTA-3’)*.* The primers amplified ~890 bp fragment (PCR sequence was sent to sequence directly, so no exact size was available) by using regular PCR at 55ºC for annealing temperature and 1 min for extension. The PCR products were digested with restriction enzyme *Hae* III, and the polymorphic bands were 480 and 410 bp for the Jagger allele and ~890 bp for the 2174 allele.


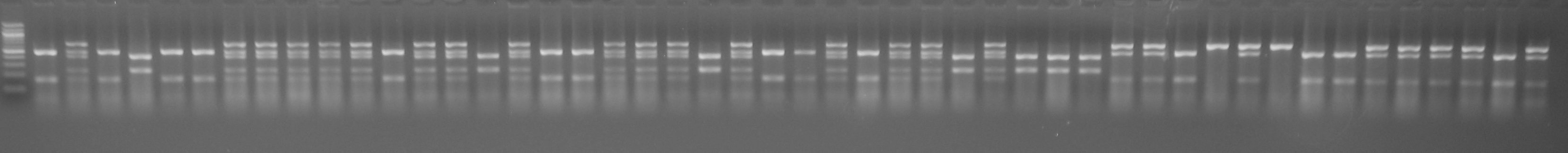


Het. Het. Jagger 2174

**Marker for *KIN***

Primers KIN5A3endF4 (5’-CTTGACTATTTTGTTGTTTATCATAATCTGTGG-3’)

KIN3endR4 (5’-GAAAACAACGACCAAATAGGCGAGC-3’) were used to map *KIN*. These primers amplified 710bp from Jagger and 722bp from 2174 annealing temperature 60℃ extension time 45sec. This marker was polymorphic between Jagger and 2174 with restriction enzyme *Rsa* I.

Jagger 2174 Het.

491 bp

342 bp


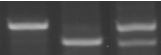


**Marker for *CBP***

Primers CBP_PromF3 (5’-CTTTATGGCATGAAAATTTTGATGTCATTG-3’)

CBP_Prom_C6R2 (5’-ACTGTATAGACTCGAAGAGAGTGCA-3’) were used to map the *CBP* gene. These primers amplified 176 bp fragment from Jagger and 167 bp fragment from 2174 using annealing temperature 60℃ and extension time 40sec. The PCR products are polymorphic between Jagger and 2174.

CBP-5A-J AGGAGTAAGGGTGTTGTGAGAACCGGGTGAACCGGGTGCTAACTCAATGGCCTGAGTTTG

CBP-5A-2 AGGAGTAAGGGTGTTGTGAGAACCGGGTG---------CTAACTCAATGGCCTGAGTTTG

***************************** **********************

Jagger 2174 Het. Het.

176 bp

167 bp


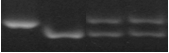


**Marker for *USPC3***

Primers USP1-C3-MF1 (5’-AAGCCACGCCTTGACCCTGTG-3’) and USP1-C3-MR1 (5’-AAAGAACACTACGTCCATGGCAATGTAATG-3’) were used to map the *USPC3* gene. These primers amplified 348bp fragment annealing temperature 60℃ extension time 50 sec. This marker was polymorphic between Jagger and 2174 with restriction enzyme *PhyCH4* III.

Jagger 2174 Het. Het.

257 bp

159 bp


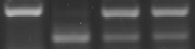


**Marker for *EX1***

Primers EX1F4 (5’*-*GCTTCTCCTGGAAGCTGCCGC-3’) and EX1C3R3 (5’-TGTCACTAAATCTCGTTAAATCCCATCATTG-3’) were used to map the *EX1* gene. These primers amplified 422bp fragment annealing temperature 60℃ extension time 50sec.This marker was polymorphic between Jagger and 2174 with restriction enzyme *Nhe* I.

Jagger 2174 Het. Het.

422 bp

289 bp


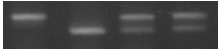


**Figure S3**


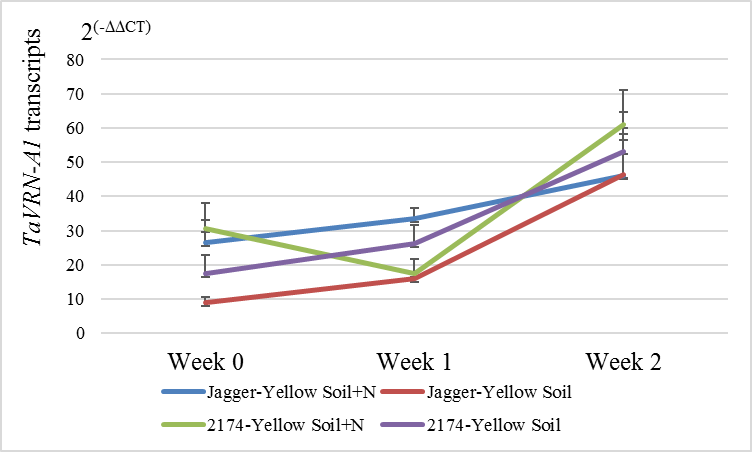


**Figure S3** **Comparison of *TaVRN-A1* expression level in Jagger versus 2174.**

The plants were grown in the same Kirkland soil which was N-deficient with normal levels of other nutrients used for the discovery of *QNue.osu-5A* and in a greenhouse, where temperature, photoperiod and moisture conditions were controlled in order to reduce interactions between genetic and environmental factors on N response. The plants were stressed for 11 weeks from planting by low soil N. The two leaf samples were collected for gene expression analysis before N was used (Week 0), and one week (Week 1) and two weeks (Week 2) after N was used. *TaVRN-A1* alleles, *TaVRN-A1a* for Jagger and *TaVRN-A1b* for 2174, did not show significant difference in transcript level. RT-PCR was used to determine transcriptional levels of leaf RNA samples by the SYBR Green PCR Master Mix, and actin was used as an endogenous control. Gene transcriptional levels are described using values calculated by the 2^(-ΔΔCT)^ method, where CT is the threshold cycle. The values represent mean expression levels (n=21, t=0.687), and the error bars indicate standard errors.

**Figure S4**


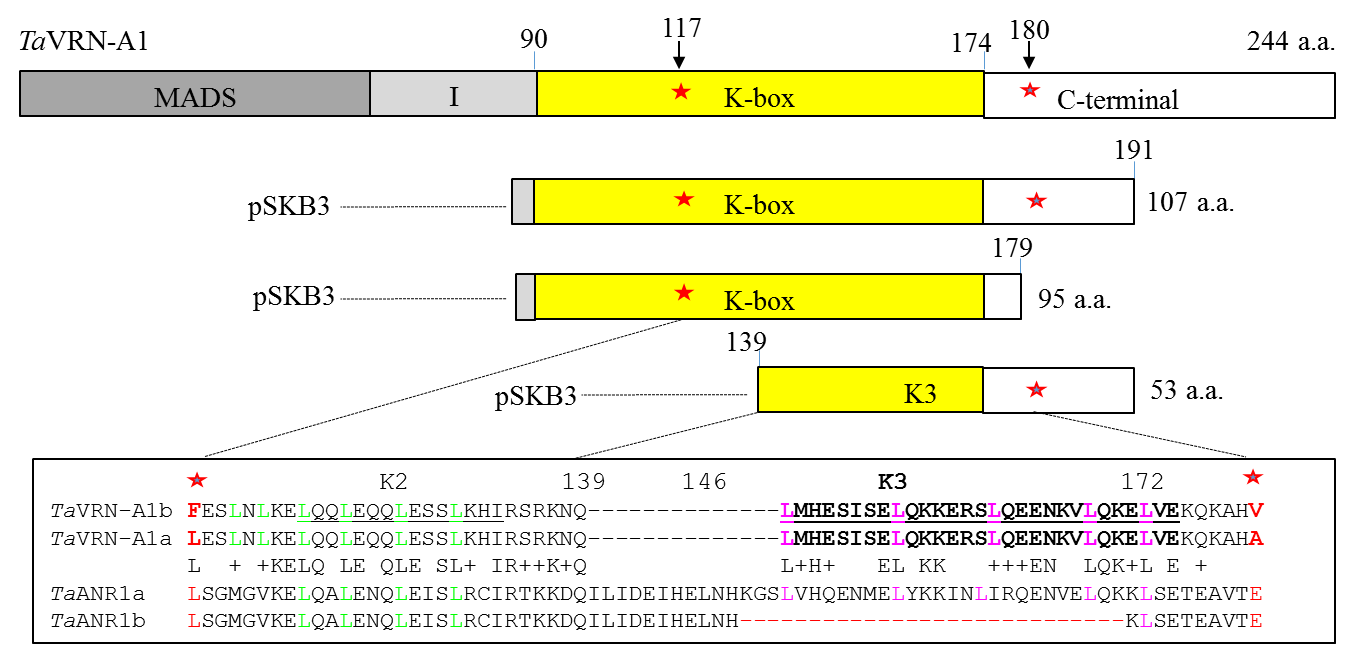


**(a)**

**(b)**

**(c)**

**(d)**

**(e)**

**Figure S4. Interacting site of *Ta*VRN-A1 and *Ta*ANR1a proteins.**

**(a)** The locations of the conserved domains in *Ta*VRN-A1 and two altered site in the amino acids are indicated in red star. **(b)** *Ta*VRN-A1 fragment covering both Leu^117^/Phe^117^ and Ala^180^/Val^180^ substitutions was expressed to test differential interactions with *Ta*ANR1a. **(c)** *Ta*VRN-A1 fragment covering only Leu^117^/Phe^117^ substitution was expressed to test differential interactions with *Ta*ANR1a. **(d)** *Ta*VRN-A1 fragment covering only Ala^180^/Val^180^ substitutions was expressed to test differential interactions with *Ta*ANR1a. **(e)** Protein sequence alignment of the *Ta*VRN-A1a from Jagger, *Ta*VRN-A1b from 2174, and *Ta*ANR1a from Jagger. Conserved Leu residues are highlighted in green in the K2 box and in pink in the K3 box. The dash in red indicate missed amino acids due to a deletion in gDNA in *TaANR1b*.

**Figure S5**

**(a)**

**(b)**

**(c)**

**(d)**

**Figure S5. The subcellular location *Ta*ANR1-YFP protein in living cells of tobacco leaves.** The *Ta*ANR1-YFP was expressed by pEG101 predominantly in a living cell. **(a)** Image of the *Ta*ANR1a protein under a fluorescent microscope with a bright filter (BF). **(b)** Image of the *Ta*ANR1a protein under a fluorescent microscope with a green filter. **(c)** Image of nucleus with 4′,6-diamidino-2-phenylindole (DAPI). **(d)** The overlay images for the alignment of the *Ta*ANR1a protein with the DAPI-stained nucleus. The scale bar in all images is 50 µm.

**Figure S6**

*Ta*ANR1 +

*Ta*HOX1a

*Ta*HOX1a

*Ta*ANR1a

*Ta*ANR1 +

*Ta*HOX1b

*Ta*HOX1b

NTC-*Ta*HOX1a

NTC-*Ta*HOX1b

Marker


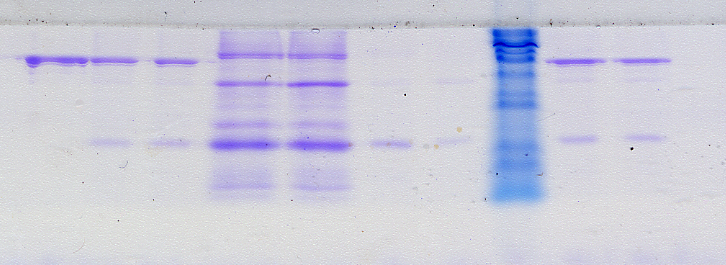


58

46

17

22

kDa

*Ta*ANR1 +

*Ta*HOX1a

*Ta*HOX1a

*Ta*ANR1b

*Ta*ANR1 +

*Ta*HOX1b

*Ta*HOX1b

NTC-*Ta*HOX1a

NTC-*Ta*HOX1b

Marker


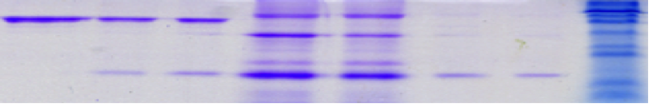


58

46

17

22

kDa

*Ta*ANR1-MBP

*Ta*ANR1-MBP

*Ta*HOX1 (1-150)-HIS

*Ta*HOX1 (1-150)-HIS

**Figure S6. *In vitro* interaction of *Ta*HOX1 and *Ta*ANR1a or *Ta*ANR1b proteins.**

*Ta*HOX1a from Jagger (20.3 kDa), *Ta*HOX1b from 2174 (20.3 kDa), *Ta*ANR1a from Jagger (60.1 kDa), and *Ta*ANR1b from 2174 (56.7 kDa) were expressed *in vitro*. The protein in the position where a star is placed is not from interaction because the protein intensity is similar as that in the negative controls (NTC-*Ta*HOX1). At least three independent replicates were performed for each of these interactions.
