# Supplementary material for: Nitrogen use efficiency is regulated by interacting proteins relevant to development in wheat
Source: Plant Biotechnol J. 2018 Jan 15;16(6):1214–26. doi: 10.1111/pbi.12864 (PMC5978868; doi:10.1111/pbi.12864)
Supplement: Supplementary file 2 — Table S1 Characteristics of the soils used for NUE. Table S2 LOD and variation of QNue.osu‐5A on 10 traits related to N utilization. Table S3 Regulatory effects of QNue.osu‐5A by N. Table S4 Wheat heading date of seven critical recombinant lines and parental lines tested in different soils. Table S5 Wheat cultivars used for determining the frequency of TaANR1 alleles. Table S6 Primers for protein–protein and protein–DNA interactions, and gene transformation. Table S7 Primers for gene expression. [file PBI-16-1214-s001.docx]

**Supplemental Tables**

**Table S1**. **Characteristics of the soils used for NUE**

Kirkland: Kirkland Silt Loam that was collected at the Cimarron Valley Research Station Research Station, OK, November 19, 2007. This soil was used in initial experiments in a greenhouse to discover the QTL *QNue.osu-5A*.

Teller: Teller Loam that was located in a field at the Agronomy Research Station in Perkins, OK, October 19, 2012. This soil was used to test seven critical recombinant lines in the field.

**Table S2. LOD and variation of *QNue.osu-5A* on 10 traits related to N utilization**

| **Traits** | **100N** | | | | **25N** | | | |
| --- | --- | --- | --- | --- | --- | --- | --- | --- |
|  | **LOD** | ***R^2^* (%)** | **A** | **B** | **LOD** | ***R^2^* (%)** | **A** | **B** |
| **Heading date**  **Chlorophyll (%)**  **Tillers per plant**  **Spikes per plant**  **Grains per spike**  **Grain weight (TKW)**  **Grain yield (g/plant)**  **Biomass (g/plant)**  **Harvest index**  **N content (%)** | 22.8  4.2  5.8  4.3  13.5  3.5  16.5  1.4  29.1  1.8 | 68.2  19.4  25.4  18.6  47.4  15.9  55.3  6.4  74.9  8.8 | 132  52.2  6  2.4  28.4  30.1  0.84  2.2  0.385  1.75 | 169  55.5  8.2  3.6  13.2  26.3  0.35  2.5  0.134  1.92 | 14.6  NS  3.1  4.8  9.1  NS  10.2  2.6  19.4  3.3 | 51.0  NS  13.9  22.7  36.4  NS  38.5  13.2  60.9  15.4 | 137  40.1  4.1  1.4  14  28.5  0.39  1.2  0.333  1.89 | 166  39.1  5.3  2.4  8.2  27.6  0.22  1.4  0.166  2.19 |

A: The Jagger allele; B: the 2174 allele.

Ten traits were characterized for two populations grown under different N levels allied to the soils. When the phenotypic traits were analyzed with the genetic map, it was found that a major QTL was associated with variation in all traits studied (Figure 1).

The populations were planted on November 18, 2007.

Fertilizers were supplied on February 13, 2008.

The tiller number was determined on March 11, 2008

Chlorophyll content was measured on March 5, 2008.

**Table S3. Regulatory effects of *QNue.osu-5A* by N**

|  | | DF | ANOVA SS | Mean Square | F Value | Pr > F |
| --- | --- | --- | --- | --- | --- | --- |
| Heading date | VRN1 | 1 43494.343 43494.343 173.06 <0.0001  1 25.566 25.566 0.1 0.7502  1 1078.415 1078.415 4.29 0.0400 | | | | |
|  | N_Rate |  |  |  |  |  |
|  | VRN1*N_Rate |  |  |  |  |  |
| Grains per spike | VRN1 | 1 4872.42 4872.42 80.11 <0.0001  1 3734.657 3734.657 61.4 <0.0001  1 899.189 899.189 14.78 0.0002 | | | | |
|  | N_Rate |  |  |  |  |  |
|  | VRN1*N_Rate |  |  |  |  |  |
| Grain yield (g/plant) | VRN1 | 1 4.5222 4.5222 97.42 <0.0001  1 3.2872 3.2872 70.82 <0.0001  1 1.0709 1.0709 23.07 <0.0001 | | | | |
|  | N_Rate |  |  |  |  |  |
|  | VRN1*N_Rate |  |  |  |  |  |
| Harvest index | VRN1 | 1 17863.089 17863.089 212.88 <0.0001  1 27.201 27.201 0.32 0.5699  1 831.631 831.631 9.91 0.002 | | | | |
|  | N_Rate |  |  |  |  |  |
|  | VRN1*N_Rate |  |  |  |  |  |
| Leaf chlorophyll content (%) | VRN1 | 1 55.015 55.015 3.91 0.0497  1 8396.615 8396.615 596.88 <0.0001  1 174.669 174.669 12.42 0.0006 | | | | |
|  | N_Rate |  |  |  |  |  |
|  | VRN1*N_Rate |  |  |  |  |  |

The one-way analysis of variance (ANOVA) was used to determine whether there was any interaction of *VRN1* representing *QNue.osu-5A* with N rate for N-related traits.

**Table S4. Wheat heading date of seven critical recombinant lines and parental lines tested in different soils****

‘*’ indicates those lines were tested in both Kirkland and commercial soils.

****** Markers flanking *QNue.osu-5A* were developed in our previous study^29^. Markers for six genes used to narrow down the *TaNUE1* region include *GT*, *STR*, *KIN*, *CBP*, *USPC3*, and *EX1.*

**Table S5. Wheat cultivars used for determining the frequency of *TaANR1* alleles**

| Allele | Source | Cultivar (with source of mapping population) |
| --- | --- | --- |
| *TaANR1a*  *TaANR1b*  (mutant) | CAP  SGP  CAP  SGP | UC1110 and CIMMYT-2 (PI 610750) (CA), Rio Blanco (ID), Zak and ID0556 (ID), P91193 and P92201 (IN), Heyne and KS01HW163-4 (KS), GRN*5/ND614-A and NY18/CC 40-1 (MN), McNeal and Thatcher (MT), Reeder/Bw-277 "R" Entry#5 and Reeder/Bw-277 "S" Plants G+LL (ND), Cayuga and Caledonia (NY), Pio 25R26 and Foster (NY), Stephens and OR9900553 (OR), Finch and Eltan (WA), Louise and Penawawa (WA), Clark’s Cream and CIMMYT III (CIGM90.250-2), Weebill and Jupateco (CIMMYT), Platte and CO940610 (CO), SS550 and PIONEER 26R46 (GA), TAM105 (NE).  Jagger, Custer, Cutter, Doans, Duster, Endurance, Fannin, Intrada, Jagalene, Jei110, Lakin, Overley, Protection, TAM 112, Danby, Deliver, Fuller, Guymon, Neosho, OK Bullet, Santa Fe, TAM 111.  IDO444 (ID), Harry and Wesley (KS).  2174, Above, Centerfield, Hatcher, Ripper, OK102, OK Field, Shocker, TAM 110, Trego. |
|  |  |  |

CAP: Coordinated Agriculture Project.

SGP: Southern Great Plains.

**Table S6. Primers for protein-protein and protein-DNA interactions, and gene transformation**

| **Primer name** | **Primer sequence (5’-3’)**^d^ | **Vector** | **Products** |
| --- | --- | --- | --- |
| VRN1(85)*Nde*IF^a^ | AGCAGCGG**CATATG**TCTGAAATTCAGGGAAACTG | pSKB3 | 85 to 191 a.a. |
| VRN1(191)*Bam*HIR^a^ | AGGAGA**GGATCC**TCAGCTGGTTTGAGGCTGAG |  |  |
| VRN1(85)*Nde*IF^a^ | AGCAGCGG**CATATG**TCTGAAATTCAGGGAAACTG | pSKB3 | 85 to 179 a.a. |
| VRN1(179)*Bam*HIR^a^ | AGGAGA**GGATCC**TCAATGGGCCTTCTGCTTCTCC |  |  |
| VRN1(139)*Nde*IF^a^ | GTGGT**CATATG**ATCAGATCCAGGAAGAACCAACTTATGCACG | pSKB3 | 139 to 191 a.a. |
| VRN1(191)*Bam*HIR^a^ | AGGAGA**GGATCC**TCAGCTGGTTTGAGGCTGAG |  |  |
| ANR-EcoRI(110)F^a^ | CGGAA**TTCTTG**ATGGGACAAGATCTTTCTGGAATGG | pMAL-c2X | 111 to end |
| ANR-BamHI(240)R^a^ | AGGAGA**GGATCC**TCATGGATGTAGTTGCAATCCTAG |  |  |
| AGLG1-NdeI-F1^a^ | AGCAGCGG**CATATG**ATGGGTCGCGGCAAG | pSKB3 | 1 to 180 a.a. |
| AGLG1(180)-BamH-R1^a^ | AGGAGA**GGATCC**CTATGTCGCCTCCAGCTCGA |  |  |
| HOX1-full-NdeI-F^a^ | AGCAGCGG**CATATG**GAGAGCGACTGCCAGTTCCTG | pSKB3 | 1 to 150 a.a. |
| HOX1(150)-BamHI-R1^a^ | AGGAGA**GGATCC**CTAGCCGCCGCAGCTAGCG |  |  |
| VRN1-BiFC2-F1^a^ | GGGGACAAGTTTGTACAAAAAAGCAGGCTTCATGGGGCGGGGGAAGG | pEG101 pEG201-YN | Full length |
| VRN1-BiFC2-R1^a^ | GGGGACCACTTTGTACAAGAAAGCTGGGTCCCCGTTGATGTGGCTCAC |  |  |
| ANR-BiFC2-F1^a^ | GGGGACAAGTTTGTACAAAAAAGCAGGCTTCATGGGGCGCGGCAAGATAGTG | pEG101 pEG202-YC | Full length |
| ANR-BiFC2-R1^a^ | GGGGACCACTTTGTACAAGAAAGCTGGGTCTGGATGTAGTTGCAATC |  |  |
| HOX1-BiFC2-F1^a^ | GGGGACAAGTTTGTACAAAAAAGCAGGCTTCATGGAGAGCGACTGCCAGTTC | pEG101 pEG201-YN | 1 to 150 a.a. |
| HOX1(150)-BiFC2-R1^a^ | GGGGACCACTTTGTACAAGAAAGCTGGGTCGCCGCCGCAGCTAGCG |  |  |
| ANR(1-180)-EcoRI F1^b^ | ATCCGGAATTCATGGGGCGCGG | pMAL-c2X | 1 to 180 a.a. |
| ANR(1-180)-BamHI R1^b^ | AGGAGAGGATCCTCATAACTCAACATT | pMAL-c2X | 1 to 180 a.a. |
| VRN1(1-180)-EcoRI F1^b^ | ATCCGGAATTCATGGGGCGGGGGA | pMAL-c2X | 1 to 180 a.a. |
| VRN1a(1-180)-BamHI R1^b^ | AGGAGAGGATCCTCACGCATGGGC | pMAL-c2X | 1 to 180 a.a. |
| VRN1b(1-180)-BamHI R1^b^ | AGGAGAGGATCCTCACACATGGGC | pMAL-c2X | 1 to 180 a.a. |
| VRN1-Ri-F1^c^ | GGACTAGTGGCGCGCCATGTATGGACAAAATTCTTG | pMCG161 | 536 bp |
| VRN1-Ri-R1^c^ | TTATAAGCGATCGCCCTAGGCTCACCATCCACGGTGGAAG |  |  |
| ANR-Ri-F1^c^ | ACTAGTGGCGCGCCCCGGACAAAAAAGGACC | pMCG161 | 310 bp |
| ANR-Ri-R1^c^ | GCGATCGCCCTAGGTGGATGTAGTTGCAATCCT |  |  |

^a^ Primers for protein-protein interactions

^b^ Primers for protein-DNA interactions

^c^ Primers for cDNA fragments that were used for RNAi constructs

^d^ Boldfaced sequences indicate the restriction enzyme sites for cloning

**Table S7. Primers for gene expression**

| **Primer name** | **Primer sequence (5’-3’)*** | **Products** |
| --- | --- | --- |
| VRN1-Exp-F1 | GAATAAAGTTCTCCAGAAGGAACTCGTG | 104 bp |
| VRN1-Exp-R2 | GCATGAAGGAAGAAGATGAAGAGCTG |  |
| ANR-MS-F2 | GAATGGGTGTCAAGGAACTGCAGG | 250 bp |
| ANR-MS-R2 | GGAGTTCTTGAATTTCGGTTAACTTCAGTCA |  |
| Actin-F2 | GGAACTGGCATGGTCAAGGCTG | 107 bp |
| Actin-R2 | CCCATCCCCACCATCACACC |  |
